# Supplementary material for: Glucocorticoid–endocannabinoid crosstalk in the ventrolateral periaqueductal gray (vlPAG) promotes pain resolution
Source: bioRxiv. 2026 Apr 29:2026.04.25.720827. Preprint. [Version 1] doi: 10.64898/2026.04.25.720827 (PMC13142447; doi:10.64898/2026.04.25.720827)

bioRxiv preprint doi: https://doi.org/10.64898/2026.04.25.720827; this version posted April 29, 2026. The copyright holder for this preprint (which was not certified by peer review) is the author/funder, who has granted bioRxiv a license to display the preprint in perpetuity. It is made available under aCC-BY 4.0 International license.

Supplementary information

Detailed Materials and Methods

Supp. Fig. 1. Repeated injections of vehicle or RIM do not affect pain thresholds in naïve rats. A. Naïve rats received daily injections of vehicle or RIM (3 mg/kg, SC) over 10 days and were then tested for mechanical thresholds (Two way ANOVA *ns* for Interaction, Drug or Time). B. Rats were also tested for thermal thresholds (Two way ANOVA *ns* for Interaction, Drug or Time).

Supp. Fig 2. Repeated injections of vehicle or RU486 do not affect pain thresholds in naïve rats. A. Naïve rats received daily injections of vehicle or RU486 (5 mg/kg, SC) over 10 days and were then tested for mechanical thresholds (Two way ANOVA *ns* for Interaction, Drug or Time). B. Rats were also tested for thermal thresholds thresholds (Two way ANOVA *ns* for Interaction, Drug or Time).

Supp. Table 1. Statistical analysis of DSI data.

Supplemental Detailed Materials and Methods.

Animals. Adult male and female Sprague Dawley rats (3-11 weeks old) were used for all experiments. All procedures were performed in accordance with the Guide for the Care and Use of Laboratory Animals as adopted by the Institutional Animal Care and Use Committee of the University of Colorado Anschutz Medical Campus.

Inflammation. Complete Freund's Adjuvant (CFA; 1 mg/ml, 0.1 ml; Sigma-Aldrich) was injected subcutaneously into the plantar surface of the right hindpaw. [39]

Drugs. WIN55,212-2 (Caymen Chemicals Ann Arbor MI, USA), SR141716A (RIM; Cayman Chemical), Corticosterone (CORT; Tocris, Minneapolis MN, USA), 11b-(4-dimethyl-amino)-phenyl-17bhydroxyl-17-(1- propynyl)-estra-4,9-dien-3-one (RU486; Tocris) were dissolved in DMSO, aliquoted, and stored at -20°C. NBQX (Tocris) was dissolved in milliQ water, and stored at 4°C. Compound101 (Cpd101, HelloBio, Bristol UK) was first dissolved in a small amount of DMSO (10% of final volume), sonicated, then brought to its final volume with 20% 2-hydroxypropyl)-b-cyclodextrin (HPCD) and sonicated again to create a 10 mM solution. PKA inhibitor (PKI, Tocris) was used directly in the internal solution in recording electrodes at 0.2 µM.

vlPAG slice preparation: Slices containing the vlPAG were prepared as previously described [34,40]. Rats were deeply anesthetized with isoflurane (McKesson, Irving TX, USA), and the brain was rapidly removed and placed in ice-cold aCSF cutting buffer containing the following (in mM): 126 NaCl, 21.4 NaHCO3, 22 dextrose, 2.5 KCl, 2.4 CaCl2, 1.2 MgCl2, and 1.2 NaH2PO4 (300 mOsm). Slices containing the vlPAG were cut to a thickness of 220 µm on a vibratome (Leica Microsystems, Deerfield IL, USA) and were transferred to a holding chamber maintained at 32°C. Slices were oxygenated with 95% O_2/5% CO_2 until transfer to the recording chamber on an upright microscope (model BX51WI, Evident Scientific, Waltham MA, USA) and superfused with oxygenated aCSF maintained at 32°C.

Whole-cell patch-clamp recordings. Voltage-clamp recordings (holding potential, -70 mV) were made in whole-cell configuration using an amplifier (MultiClamp 700B, Molecular Devices), sampled at 2 kHz, and digitized at 5 kHz with the Axon Digidata 1550B (Molecular Devices, USA) using Clampex 11.0.3 software (Molecular Devices, USA). Patch-clamp electrodes were pulled from borosilicate glass (diameter, 1.5 mm; WPI) on a two-stage puller (Narishige). Pipettes had a resistance between 2.5 - 4 MΩ and were filled with an intracellular pipette solution containing the following (in mM): 140 CsCl, 10 HEPES, 4 MgATP, 3 NaGTP, 1 EGTA, 1 MgCl2, and 0.3 CaCl2 (pH 7.3, 290–300 mOsm). QX314 (100 µM) was added to the internal solution for evoked IPSC (eIPSC) experiments to reduce action potentials in the recording cell. Access resistance was continuously monitored. Recordings in which access resistance changed by 20% during the experiment were excluded from data analysis. A bipolar stimulating electrode (FHC, Bowdoin ME, USA), placed into the vlPAG approximately 200 µm from the recording electrode, was used to deliver 2 ms pulses of 100 µA to 10 mA to evoke IPSCs. A junction potential of 5 mV was corrected during recording. GABAergic eIPSCs were isolated in the presence of glutamate receptor antagonist (NBQX; 5 µM). In experiments using exogenous cannabinoid agonists/antagonists, or GR agonists/antagonists, only one neuron was recorded per slice. After each experiment, the lines were washed with 70% ethanol and then rinsed with milliQ water.

bioRxiv preprint doi: https://doi.org/10.64898/2026.04.25.720827; this version posted April 29, 2026. The copyright holder for this preprint (which was not certified by peer review) is the author/funder, who has granted bioRxiv a license to display the preprint in perpetuity. It is made available under aCC-BY 4.0 International license.

Depolarization-induced suppression of inhibition. After obtaining stable eIPSCs, a protocol for DSI collected 2-3 eIPSCs for baseline measurement, followed by a brief depolarizing step (5 s at +20 mV; [41]) before returning to the holding potential. eIPSCs were evoked at 0.2 Hz for 60 s following the depolarizing step, and normalized to the average baseline eIPSC amplitude. Cells were grouped into "DSI" or "No DSI" with DSI defined as a minimum of 10% inhibition of eIPSCs.

Endocannabinoid analysis. Quantification of 2-AG and AEA level were performed in the vlPAG, the RVM and the injured paw tissue of naïve and CFA-treated rats. Tissues were removed, quickly frozen in liquid nitrogen and stored at -80ºC degrees until analysis. Approximatelty 50 mg of tissue powder were placed into 1000 µL of acetonitrile/methanol (1/1, v/v) containing an isotope labeled internal standard mix on a scale to record the actual tissue weight. Samples were shaken and centrifuged at 25000xg, 4ºC for 15 minutes. Supernatants were transferred to HPLC sample vials and analyzed using HPLC mass spectrometry as previously described [42-44]. Following shaking and centrifugation, the supernantant was analyzed as described above and protein pellet was dried and reconsituted in detergent buffer to determine the protein content.

Measurement of plasma CORT levels. Plasma CORT levels were determined using trunk blood immediately after anesthesia at the time of slicing for electrophysiological recordings in the morning to avoid variation due to circadian rhythm. Blood fractionation was achieved by spinning the blood samples at 14000 rpm for 20 min using a centrifuge (Model 5418R, Eppendorf). Supernatant containing plasma was rapidly collected and stored at −80°C until subsequent analysis. All samples were analyzed using the commercially available enzyme-linked immunosorbent assay (ELISA) CORT kit (#EIACORT, ThermoFisher Scientific) according to the manufacturer's specifications.

Behavioral studies. **Mechanical nociception** was assessed using an electronic von Frey (Ugo Basile, Gemonio, Italy). Prior to the experiment, rats were acclimatized to a Plexiglas chamber for 15 min (grid: 0.5 cm × 0.5 cm; box: 10 cm × 10 cm × 15 cm) on a raised-mesh metal platform. Responses from each hind paw were measured three times each, and the mean for each paw was calculated as the paw withdrawal threshold (PWT). Baseline responses were measured before CFA injection (D0) and at various time points. All measurements were performed by the same person, blinded to treatment.

Thermal nociception was assessed using the Hargreaves apparatus (Plantar Test, Ugo Basile, Gemonio, Italy), which measures paw withdrawal latency (PWL) to radiant heat stimuli. Rats were acclimatized in singular Plexiglas chambers (10 cm × 10 cm × 15 cm) on a glass platform for 15 min. The radiant heat source was applied to the center of the plantar surface of each hind paw with 2 min intervals between each application, and PWL determined as time to retraction or licking of the hind paw. To avoid tissue damage, a cut-off of 25 seconds was used. All trials were performed three times for each hind paw, and the average for each hind paw was calculated as the PWL. All measurements were performed by the same person who was blinded to the treatment.

vlPAG microinjections. To assess the central effect of RU486 on behavior, rats performed mechanical and thermal nociception tests as previously described, then the animal were anesthetized with vetflurane (3%, Piramal, PA USA) using a R500 Small Animal Anesthesia Machine (RWD, Sugar Land, TX USA). Unilateral microinjection of RU486 (3 µg/kg dissolved in 10% DMSO in saline, 0.4 µl in one minute) into the vlPAG was performed using a stereotaxic apparatus (NeuroSTAR, Germany). The injection cannula was left in place for 2 min to minimize backflow. Immediately after microinjection, wounds were closed and animals were allowed to recover for 5 min. Animals were subjected to the behavioral testing protocols 15, 30 and 60 min post injection.

Statistical analysis In all electrophysiological experiments, each dataset included recordings from at least two male and two female rats. For DSI experiments, two separate time windows were analyzed: the average of the first five eIPSCs (0–25 s) following depolarization, and the average of the subsequent five eIPSCs (30–50 s). All analyses were conducted in Graphpad Prism (Graphpad Software). Values are presented as the mean ± SEM, and all data points are shown in bar graphs to illustrate variability. Statistical comparisons were made using t-test or ANOVA, as appropriate. In all summary bar graphs for electrophysiology experiments, each dot represents an individual cell while the numbers in the bars represent the animal number. When post hoc analysis was appropriate, multiple-comparisons tests were performed and specified in the figure legends. Statistical significance was defined as $p < 0.05$.

bioRxiv preprint doi: https://doi.org/10.64898/2026.04.25.720827; this version posted April 29, 2026. The copyright holder for this preprint (which was not certified by peer review) is the author/funder, who has granted bioRxiv a license to display the preprint in perpetuity. It is made available under aCC-BY 4.0 International license.

Supplemental figures

Sup Fig1

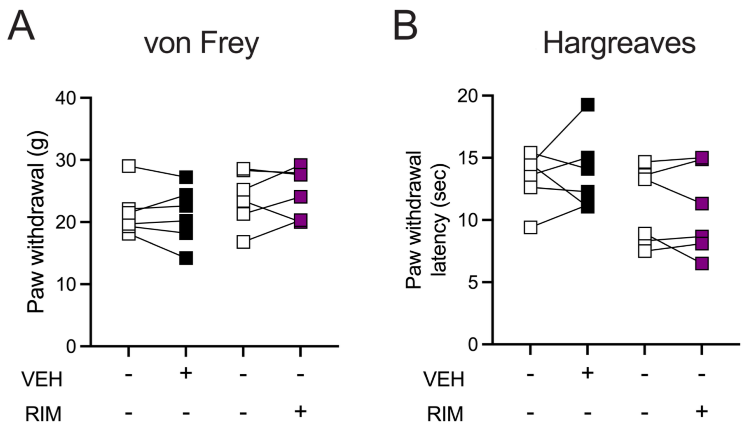

Sup Fig 2

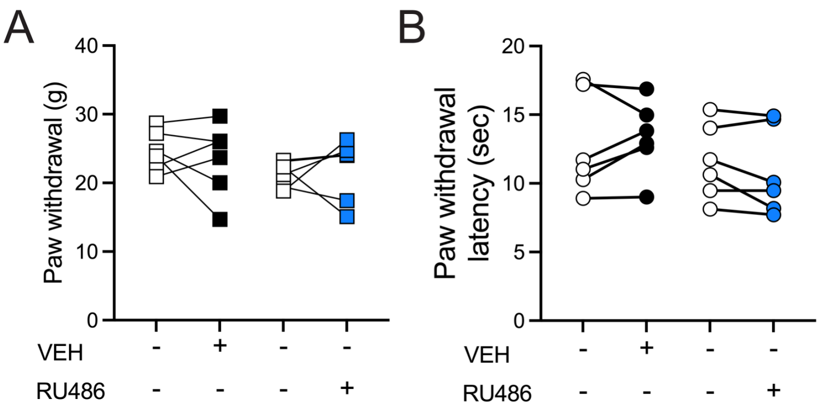

Supplement: Supplement 1 [file NIHPP2026.04.25.720827v1-supplement-1.pdf]
